# A Systematic Approach to Agile Development in Highly Regulated Environments

**DOI:** 10.1007/978-3-030-58858-8_12

**Published:** 2020-08-18

**Authors:** Alexander Poth, Jan Jacobsen, Andreas Riel

**Affiliations:** 6 IT University of Copenhagen, Copenhagen, Denmark; 7 University of British Columbia, Vancouver, BC Canada; 8 Volkswagen AG, Berliner Ring 2, 38436 Wolfsburg, Germany; 9 Volkswagen Financial Services AG, 38112 Brunswick, Germany; 10 Université Grenoble Alpes, CNRS, Grenoble INP, G-SCOP, 38031 Grenoble, France

**Keywords:** Software development management, Agile software development, Regulation compliance, Large scaling agile

## Abstract

For established domains within highly regulated environments, a systematic approach is needed to scale agile methods and assure compliance with regulatory requirements. The presented approach works adequately in small agile teams – independently of the underlying method such as Scrum, Kanban, etc. – and is scalable to more and bigger teams or even entire subsidiaries. It is based on a compliance and a quality risk dimension respectively. Both dimensions are needed to fit regulatory requirements in our finance example with more than 100 developers in one subsidiary.

## Introduction

Established industry sectors are more or less regulated. Less regulated sectors solely have to incorporate basic requirements like European Union regulation, i.e. the General Data Protection Regulation (GDPR) [1], and/or national requirements such as the German Commercial Code (HGB) [2]. In highly regulated sectors however, products and services have to comply with further extensive standards and regulations. The financial sector, for example, has to fulfill regulations imposed by the EU countries’ national supervisory authorities, as well as Minimum Requirements for Risk Management for financial institutions (MaRisk) in Germany [3]. Many regulations are domain-specific like medical, finance or automotive. However, regulations have some common aspects like quality assurance evidences for verification and validation which demand a more or less stringent traceability and risk management [4].

Our research objective is to design a framework that can be used to derive a specific compliance guideline offering as much autonomy to agile teams as possible by fitting the required specific regulations of the product or service with its organization. In large organizations, specific organizational units have to be aligned with specific compliance requirements. To support this specificity, the approach shall be generic by design. This will enable scaling the approach into different organizations and their units. As for evidences for the effectiveness of the framework, we want to meet the following three core requirements. First, the external confirmation by audits with focus on compliance shall be facilitated. Second, the delivery of the demanded business value shall not be hampered and remain an essential part of the outcome flow. Third, the framework shall be adaptable to new regulations over time.

## Related Work and Methodology

A huge body of documentation exists to handle regulation and compliance. However, these works mostly focus on a specific solution or aspect within the respective domain. This leads to partial [5] and inconsistent [6] agile adoptions [7] like ScrumBut. Examples for agile development in regulated domains are [8] for safety related products, [9] for the medical, and [10] for the finance domain. However, it is difficult to find a generic practical framework for regulated domains.

The framework presented here was developed following the design science research approach [11], demonstrating the framework’s application in a case study in the financial domain. The framework’s general applicability is assured by design thanks to its independence from any specific regulation. Furthermore, it is adoptable by design to different business domain specific demands in large organizations to scale into their units.

## Scaling Conformity to Regulations via Levels of Done

The development process has to address two dimensions. The domain dimension handles the organizational and procedural compliance requirements. It has to assure that the compliance requirements be fulfilled at least at the latest required point in the product or service life cycle. Earlier assurance of regulatory requirements is possible and a part of the team’s self-organization. The product specific dimension helps teams identify and realize their product specific quality-risk requirements. Within this dimension, the team handles product or service specific quality-risks in a structured and transparent manner to assure an adequate risk management. For handling the product specific quality risks, we use the Product Quality Risk (PQR) [12] approach, which focusses on quality risks implied by the specific market chances and opportunities of each service or product. PQR guides the teams from a systematic identification of specific service and product quality risks, and helps them define adequate mitigation actions.

To leverage a lean and agile development process, which teams can apply outcome-specific refinements to, only a minimum predefined framework shall be set while still assuring a systematic handling of the team’s refinement work. The process outcome’s value is assessed by its (inherent) quality risks. Systematic product or service quality risk identification and handling proposed in [12], can be used to assure that the development process does not lose outcome focus. In [5], the product capabilities and features are used to derive the product specific quality risks. Based on the identified and prioritized quality risks, adequate mitigation actions are scheduled during the development to ensure a compliant and high quality outcome.

To assure that product teams incorporate both dimensions just in time, we propose a Levels of Done (LoD) approach. LoD are an enriched variant of the Definition of Done (DoD) of Scrum that is aligned with requirements [13] at defined milestones in the development process. The LoD approach applies the concept of boundaries [14] beyond the sprint time-box between Definition of Ready (DoR) and DoD to all take-overs in a value chain. This makes it simple and independent from any specific agile approach based on sprints, as well as sufficiently generic to adapt to different regulation domains with the specific check-points they require. This is necessary to fulfill a systematic product and process quality approach demanded by most quality related standards, as well as to allow agile scaling while staying effective [15].

## The LoD-PQR Approach

While in a traditional compliance scope, the software development life-cycle is clearly defined by a comprehensive set of fixed requirements and deliverables prior to project start, we propose the following four steps to define LoD in agile environments:

**Identify all relevant regulations and** standards of your enterprise for compliant products and/or services.

**Identify how many stages you have** for product development via a Kanban board.

The Kanban board helps to identify handover-points in a work stream. These points are the most relevant for LoD.

According to Conway’s law [16], the structure of an origination drives their outcomes. Therefore, alignment of the “planned” outcome architecture with the organization shall be considered. This should also drive future changes to an existing LoD to support the transformation in a pull-fashion. The LoD does not refine the internal team organization between two stages. The teams can apply their preferred agile approach like Scrum, Kanban etc. in their self-organized working flows to fit the next stage.

**Enabling teams to choose the most effective ways** to comply with regulatory relevant outcomes by mapping them to the stages of the Kanban board.

**Fig. 1.**
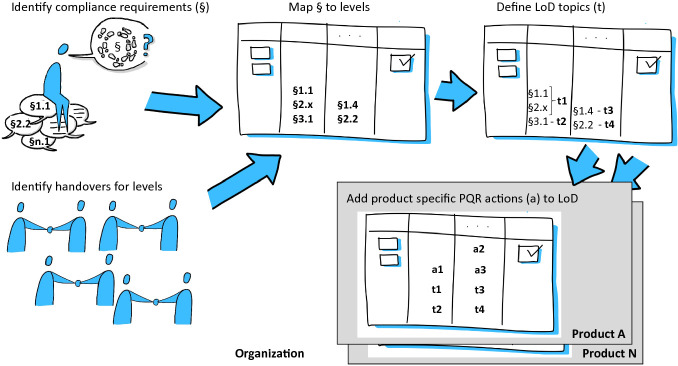
Schematic picture of a practical LoD-PQR method application scenario.

A transparent traceability from the regulation to the LoD will facilitate regulation adoption. However, finding adequate implementations should be delegated to the team to give them freedom to find solutions that fit into their particular context. The openness about how to reach the outcomes give the teams the autonomy to work as it is best for their specific demands and the mastery (responsibility) about their implementations. The traceability from the external requirements to their internal representations – the topics in Fig. 1 – shall be established to avoid interpretations by missing “root” and to avoid non-value adding activities in a lean context.

**Reduce the outcomes of “chains”** to the last outcome for a shorter list.

To optimize the LoD, chains of dependencies can be reduced to the latest outcome. For example, a separate test protocol is not needed if the test result log and protocols are saved as part of the comprehensive deployment-log and stored in an auditable way. This is covered by an underlying internal control system.

**Provide additional information** about practices and work instructions about outcomes for assisting the teams. To help the teams for a fast instantiation, a practice collection can be provided sharing of experiences across the organization. If a new practice is identified, it will be added to the practice collection to leverage continuous improvement and replacement of outdated practices.

**Add the PQR dimension** to assure that products and services have a comprehensive quality approach. To derive systematically the specific PQR a self-service kit for the teams is recommended as described in [17]. While the LoD covers only formal regulation requirements, the PQR method handles business risks related to deliverables by quality related mitigation actions as described in [12] and [17]. These mitigation actions are mapped to the corresponding stages and handled by the teams. Based on the regulation and quality risk dimension, a holistic quality management system can be established. Figure 1 shows how the actions fit together in a product team specific instantiation. It visualizes the instantiation of the 4 LoDs, the product team specific PQRs actions (a) on top of the organization-wide valid LoD topics (t), as well as the numerous product checks.

**Fig. 2.**
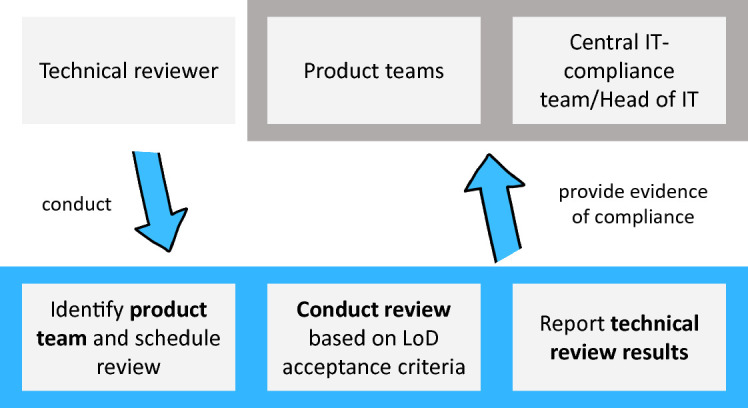
LoD compliance process and involved stakeholders.

The LoD-PQR approach is easily repeatable for the iterative and incremental development in agile product teams. It also foresees cross-team reviews conducted by technical reviewers (IT experts) providing evidence of compliance with the LoD. Quality standards covered in the reviews include: architecture, code quality, PQR, security, documentation, etc. Every topic has its own LoD acceptance criteria. Depending on the technical review result, the accountable role (e.g. Head of IT) grants technical approval for the product release (Fig. 2).

One difference to a DoD is that the latter is typically defined by the team, while a LoD is given by the organization to a team, and team-specific parts are defined via the PQR with a product or service focus. A second difference is that a DoD addresses aspects which are handled by the team, while the LoD-PQR approach ensures an end-to-end view for a delivery of a product or service. Furthermore, the DoD is checked by the team as a kind of a self-commitment, while the LoD is typically checked and ensured by team external reviews initiated by the organization’s compliance.

For the review and approval process as well as the LoD, internal criteria shall be derived. The control owner shall establish a monitoring on the whole process against these criteria (via preventive gates and/or detective post-checks) in order to conduct appropriate actions depending on the level of conformance and control effectiveness.

Derivations to the LoD shall be assessed and tracked to sign-off by the risk owners. Teams “pull” experts for specific standards for support in case of new or special issues. Any regulation changes shall be integrated into the LoD as soon as possible and all teams have to ensure to fulfill the current version as soon as possible. Teams can autonomously set synchronization points in case of inter-team dependencies. The time span between the different levels of the LoD in a team mostly depends on the team’s delivery frequency, and is independent from a team’s delivery cycle duration. Some teams need weeks, others months.

## Case Study: Instantiation, Deployment and Its Limitations

The Volkswagen Financial Services AG Digital Unit Berlin (DU) identified four stages for their LoD (cf. Fig. 1). First, the business takes over the stories into the team. Second, the team implements the requirements according to compliance for security etc. Third, the product is checked for compliance and business process integration. Finally, the product’s functionality is verified during operation. The last stage is interesting for the handover in cases were no DevOps is applied.

The identified regulations and standards for the financial domain are defined by the European Union and are instantiated by German governance and regulation institutions like the MaRisk, BAIT [18] or GDPR. As shown in Fig. 1, a key input to LoD was the experts’ collection of LoD-relevant requirements. They derived them from the relevant regulations and collected them in a central document. Subsequently they mapped similar requirements and merged them. They integrated requirements addressing the development process (e.g. independent checks from the business of IT systems in BAIT requirement 41) into the LoD design. These requirements from the *identify compliance aspects* of Fig. 1 have an impact on the team’s organization and their interfaces. Hence, regulations impact organization setup and team handovers (*identify handovers for levels* in Fig. 1), in as described in Conway’s law. In the given context, this happened for the acceptance testing by the business which is realized in an independent stage in *map § to levels* in Fig. 1. Based on this, all teams have to instantiate this regulation implementation before they can *add product specific PQR actions* in the last step in Fig. 1. Another example is the regulation requirement about systematic requirement documentation of the BAIT requirement 37 “Requirements for the functionality of the application must be compiled, evaluated and documented in the same way as for non-functional requirements.” This regulation requirement about requirements is handled in the LoD’s first level with the task to refine requirements based on the recommendation to align stories on the INVEST criteria [19]. INVEST stands for Independent, Negotiable, Valuable, Estimable, Small and Testable. The recommendation is given to establish a kind of state of the art for requirements documentation however teams have the option to substitute the recommendation with another more adequate method for the product or service context. Furthermore the BAIT 37 requires “The organisational units shall be responsible for compiling and evaluating the requirements.” which leads to assign it to the LoD’s first level – responsibility for this level is by the business product owner - and not to the second level with IT responsibility. Both examples show that in a regulated finance environment one team have to has hand-over points which leads to at least three levels of done to be compliant to the BAIT.

Preventive checks of the LoD’s correct application are conducted before a productive deployment, while detective compliance check are done after deployment. To assure LoD compliance, the DU adopted the approach from Fig. 2 with some refinements for adequate review sampling and time (pre- or post-deployment). To reduce the direct effects of the LoD procedures on team level, the objective is to reduce the pre-deployment checks, which interrupt the delivery workflow of the team for a compliance task. However, each team has to ensure that in an audit, all relevant artifacts and evidences are available to demonstrate a compliant delivery.

The LoD of the DU has been developed by a cross-functional team. The team incorporated experts from the headquarters compliance, headquarters security, business and development teams, as well as external experts from the Volkswagen AG. Reflections with external consults (agile coaches, auditors etc.) were done cyclically too. Throughout the development period of almost one year, the team allocated approximately 6–7 experts. The initial application (evaluation) in the first teams was done with facilitation by the expert team. After small enhancements and the positive feedbacks of the early adopter teams, a LoD Community of Practice (CoP) was established. This was useful to ensure that the scaling to all teams can be made efficient and quick. The experts are limited resources and in the CoP the teams can help each other too – this helps to reduce bottlenecks by the experts who were focusing on the new issues and questions.

In the last 3 years we established and enhanced the approach for more efficient delivery and to regulation updates with the Scrum masters and the teams. Currently more than 100 developers are working with the LoD-PQR approach, and further locations and organizational units are in the adoption phase.

The application to the DU financial case revealed the following limitations of the LoD-PQR approach with respect to the corporate governance having to assure

- The correct outcomes for the compliance requirements, as well as
- The expected deliverable which creates the customer/user value;
- The update of the LoD by the regulation experts;
- The update of the PQR by the product or service experts.

These limitations are partly addressed by the review procedure (Fig. 2), which however generates a base workload scaling linearly with the delivery frequency of the products and services. To reduce this linear correlation of reviews to deliveries, a team maturity approach can be established. Higher team maturity leads to more autonomy and thus reliefs the team from having mandatory pre-deployment LoD-triggered technical reviews by team-independent reviewers.

## Discussion and Conclusion

The LoD-PQR approach addresses the demand for a generic approach to handling regulation requirements and product specific quality management in an agile environment. While we have shown the generic LoD-PQR method application to the European finance domain, other domain specific requirements would need to be identified, e.g. for the DO-178 (avionics safety) or ISO 26262 (automotive safety). However, the amount of regulation requirements in finance was lower than initially expected, approximately 50 with direct impact to the software development. The product specific PQRs strongly depend on the outcomes, however the workload which can be handled by a team is a “limiting factor”.

The acceptance of our methodology within the agile-teams was encouraged by the committed degree of freedom. In our case, we have witnessed that implementing the LoD-PQR approach supported the teams to navigate through the complex compliance requirements in our domain in a lean way (conformity). Our approach enabled the product teams to realize efficiency by design and to share techniques how to implement compliance requirements in an uncomplicated way. Besides, the genuine learning character of the LoD-PQR approach leads to streamlined development processes of the approach itself, leading to a positive impact on process performance.
